# Extraction bradycardia: a pilot case-crossover study

**DOI:** 10.1186/1746-160X-9-29

**Published:** 2013-10-17

**Authors:** Ashkan Rashad, Ralf Smeets, Madiha Rana, Behnam Bohluli

**Affiliations:** 1 Department of Oral and Maxillofacial Surgery, University Medical Center Hamburg-Eppendorf, Martinistrasse 52, Hamburg 20246, Germany; 2 Department of Differential Psychology and Psychological Assessment, Helmut-Schmidt-University, Hamburg, Germany; 3 University of the Federal Armed Forces, Hamburg, Germany; 4 Craniomaxillofacial Research Center, Azad University of Tehran, Tehran, Iran

**Keywords:** Tooth extraction, Trigeminocardiac reflex, Bradycardia, Heart rate

## Abstract

**Purpose:**

Significant vasovagal reaction is one of the untoward events in the course of simple extractions. The present study then aimed to record the patients’ heart rate during the extraction procedure.

**Materials and methods:**

Informed consents were obtained in advance. Patients were placed in the dental chair and their heart rate was measured before /and prior to the anesthetic injection, during, and after dental extraction on a pulse oxymeter device. Data were analyzed using paired t-test.

**Results:**

Sixty one patients were included. The mean heart rates of these patients prior, during, and after extraction were 88, 86 and 81, respectively. Two by two comparisons showed a significant decrease in the mean heart rate during extraction compared to the baseline and also after extraction compared to both before and during extraction (*p* < 0.05 for all three).

**Conclusions:**

Despite the presence of sufficient local anesthesia and performing the extraction with the least trauma, a significant decrease in heart rate is evident.

## Introduction

It has been discussed that tooth avulsion is the most critical point of the extraction procedure in terms of hemodynamic alterations [1,2]. Transient Loss of Consciousness (TLOC) also known as Vasovagal Reflex (VVR), which in this case is most probably of non-cardiac subtype, has been reported to occur upon extraction maneuvers. More recently, Trigeminocardiac Reflex (TCR) has also been suggested as the possible mechanism of critical hemodynamic alterations upon extraction [3,4].

Different authors have found that in healthy normotensive patients, slight elevations of Systolic Blood Pressure (SBP) and Diastolic Blood Pressure (DBP) occur up to the moment of extraction followed by a decreased SBP and DBP to levels even lower than those recorded at baseline [1,2]. This is thought to be attributable to the release of endogenous adrenaline as a reaction to the noxus [5,6]. Generally, Heart Rate (HR) and Blood Pressure (BP) and other clinical hemodynamic alterations throughout the extraction procedures are also a factor of patient age, gender, pain perception, psychological factors, and systemic conditions as well as local anesthetics, and the procedure itself among other factors [7,8].

While clinicians have been advised to take into account monitoring and managing the hemodynamic alterations during dental procedures [5,6,9-13], the body of literature on the topic remains to be inconclusive and scarce [1,2,4]. For example, while VVR has been suggested as the cause of dental office syncope during extraction, studies have not always shown the biphasic nature of the reflex [1,4]. Therefore, in the present study, the authors have attempted to revisit the extraction bradycardia in a prospective cohort of normotensive, otherwise-healthy patients and discuss the findings within the contemporary literature in the context of the current (pathophysiological) knowledge.

## Materials and methods

### Patients

The study protocol was peer reviewed and approved by Azad University of Medical Sciences, Tehran/Iran. This prospective case-crossover study included patients referred to Oral & Maxillofacial Surgery Department of above mentioned university for simple extraction of one posterior mandibular tooth during late 2011. Signed informed consents were obtained chair side in advance. The guidelines of the Helsinki Declaration have been followed for this investigation. Excluded were the patients with a history of cardiovascular or pulmonary disease and those for which more complicated surgical procedures like flaps, decoronation, or fragmentation of root was followed. Also included were only the fully erupted third molars of level A depth and Class I available space [2].

### Methods

The demographic data of all patients were then recorded in questionnaires through interviews. Pulse oxymeter (Nellcor OXIMAX IM-N-550, Tyco Healthcare Group LP, Pleasanton, USA) was used to record the HR of all patients prior to the injection of local anesthetic. Up to two cartridges of 4% Articaine with 1:100.000 epinephrine (Ultracaine DSF®, Sanofi-Aventis GmbH, Berlin, Germany) was used through conventional inferior alveolar nerve injection to obtain local anesthesia. HR was also recorded during extraction (lowest HR detected) and also immediately afterwards. All the values were recorded in the same questionnaire used for each patient. Dental explorer was used to ensure proper anesthesia before extraction. All the extractions were performed by one oral and maxillofacial surgeon and care was exerted to avoid excess trauma or inadequate anesthesia. Extraction of the erupted posterior mandibular teeth were conducted similar to usual intervention in order to simulate common extraction settings.

### Statistics

PASW Statistics 18 (IBM corp., NY, USA) was used for data analysis. The mean HR (MHR) and standard deviations (SD) were calculated and paired *t*-test was used to detect possible differences between the MHR values prior, during and after the injection of local anesthetic. Differences were considered significant at p < 0.05.

## Results

### Demographics

A total of 61 patients were included into the study based on simple consequential sampling. Included were 27 (44.3%) males and 34 (55.7%) females, ranging in age from 18 to 59 years (31.1 ± 9.9). Most of the patients were 21 to 30 years old (42.6%). Extracted were 61 mandibular molars including 34 (55.7%) third, 14 (23%) second, and 13 (21.3%) first molars.

### Heart rate and age

Table 1 summarizes the mean ± standard deviation of the heart rate prior, during and after extractions according to the age groups. No statistically significant differences were found in the MHR between all different age groups before (*p* = 0.70), during (*p* = 0.88), or after (*p* = 0.84) the extraction.

**Table 1:** The mean ± standard deviation of the measured heart rate values prior, during and after extractions according to the age groups

**Age groups**	**Frequency**	**Before**	**During**	**After**
15-20	3	86.7 ± 14.6	88.7 ± 3.5	80 ± 8.0
21-25	13	86.3 ± 9.5	84.6 ± 9.5	80.5 ± 8.8
26-30	13	87.1 ± 9.5	82.4 ± 10.4	78.7 ± 10.1
31-35	8	94.4 ± 21.5	90 ± 17.8	83.2 ± 9.3
36-40	9	92.1 ± 14.3	82.2 ± 16.5	85.5 ± 14.3
41-45	10	88 ± 18.4	84.5 ± 17.1	79.8 ± 15.4
46-50	1	102	100	96
51-55	2	93 ± 21.2	90 ± 21.2	85.5 ± 17.7
56-60	2	85 ± 7.1	84 ± 11.3	80 ± 14.4
Total	61	88.4 ± 14.1	86.1 ± 13.4	81.4 ± 11.4

### Heart rate and gender

Table 2 summarizes the mean ± standard deviation of the pulse rate prior, during and after extractions according to gender. The MHR values after extraction were statistically lower than those of during and before extraction in both males and females (*p* = 0.000). The only exception was the MHR decrease of females during the extraction compared to before it (*p* = 0.97).

**Table 2:** The mean ± standard deviation of the measured heart rate values prior, during and after extractions according to the genders

**Gender**	**Frequency**	**Before**	**During**	**After**
Male	27	87.2 ± 13.8	85.7 ± 13.5	80.0 ± 11.7
Female	34	89.4 ± 14.5	88.0 ± 13.1	82.6 ± 11.2
Total	61	88.4 ± 14.1	86.1 ± 13.4	81.4 ± 11.4

### Heart rate globally

**Figure 1:**
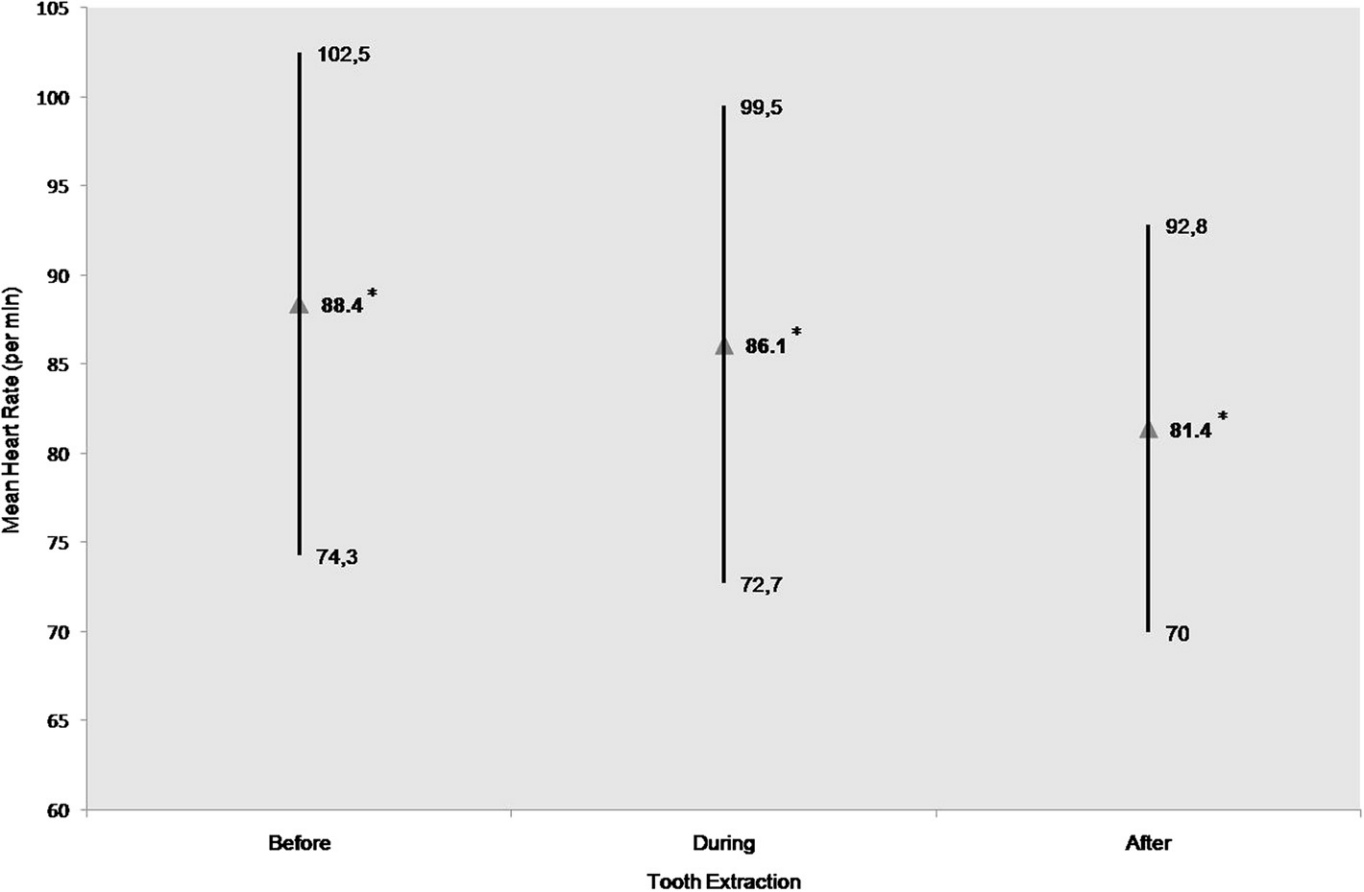
**Distribution of mean heart rate values (per min) for total patients (n = 61) before, during, and after tooth extraction.** * illustrates significant differences between all 3 measured mean heart rate values.

The MHR values of the study population showed statistically significant decreases between the three measurement attempts meaning the MHR (*p* = 0.000 for each of the three comparisons) (Figure 1).

## Discussion

Included were 61 simple extractions of the posterior mandibular teeth for which HR was recorded prior, during, and after extraction and compared. It was shown that MHR decreases significantly from the baseline both during and after extraction. The global decrease of the MHR is consistent to the findings of others [2]. Alemany-Martinez et al. [2] have measured the MHR at nine points during the extraction procedure namely at baseline, 1 minute after anesthesia, 4 minutes after anesthesia, incision, osteotomy and/or tooth sectioning, completion of extraction, start of suturing, end of the surgical procedure, and removal of the surgical drapes. In the present study, there were no need for incisions, tooth sectioning, and suturing. Therefore, the comparisons are only possible between the baseline values and those of the peri- and post-extraction points.

Even so the global findings of the present study are consistent to those of the Alemany-Martinez et al. [2] for MHR values of peri- and post-extractions, they have recorded an increased HR between the baseline and during extraction. In their study, a gradual increase of MHR from the baseline to immediately before extraction is detected. Consistent to the findings of Paramaesvaran and Kingon [1], the HR alterations of the present study over time and through the extraction procedure have been significant. They investigated the BP and HR during the extraction procedure of 30 patients. In their study, a transient bradycardia upon local anesthesia administration has been also reported [1]. It should be noted that despite the presence of the same finding in the study of Alemany-Martinez et al. [2], the transient bradycardia is actually concurrent with elevated SBP and DBP.

Stimulation of the cranial nerve V anywhere throughout its course, let it be the three peripheral branches namely maxillary mandibular or ophthalmic branches, has been shown to be associated with an abrupt decrease of HR which is thought to be as significant as about 20% in central type of the reflex observed during neurosurgical and other skull base procedures [14-17]. After some first works by Guiesepe & Arscher in 1908, this reflex, called trigeminocardiac reflex (TCR), was introduced and described in detail by Schaller et al. since 1999 [18]. The afferent pathway of the TCR consists of short and long ciliary nerves, which relay the impulses to the ciliary ganglion and then to the Gasserian ganglion [14,18]. The afferent pathway is completed in the sensory nucleus of trigeminal nerve in the floor of the forth ventricle [14,18]. Small internuncial nerve fibers of the reticular formation connect the afferent to the efferent pathway. The efferent pathway originated from the motor nucleus of the vagal nerve and sends depressor fibers to the myocardium thus complementing the reflex arc [14,15]. The patients in our present series did not show a biphasic alteration of HR. In other words, the decrease in HR is not preceded by a preliminary increase. Hypothetically speaking, the findings of the present study further challenge the belief that VVR is responsible for the hemodynamically mediated accidents and emergencies. Although, no cases of VVR were seen in the present study, the possibility of developing more severe bradycardia and subsequently syncope especially in patients with poor stress management and insufficient local anesthesia should not be overlooked [3,4]. On the other hand, the pattern of HR alterations of the present study is consistent to our recent modification on the widely accepted description of TCR. TCR, as the name implies, includes any cardiac reflex triggered upon the stimulation of the trigeminal nerve anywhere throughout its course. Clinically, however, TCR might be best described as sudden onset of bradycardia (not necessarily a 20% decrease as stated in the original definition) [15]. upon the stimulation of the trigeminal nerve, anywhere throughout its course [16,17].

The present study stands among the first to have described a peripheral TCR during teeth extraction [3,4]. It is best known that peripheral TCR is somewhat different to central TCR [18], a fact that is again underlined by the present study. Hemodynamic monitoring during extractions is therefore advisable especially when the surgical procedure is expected to be traumatic or involve more soft and hard tissue manipulations as the surgeon will be able to readily detect untoward events. This is even more important when the patient’s psychological condition makes such control desirable to optimize safety [2]. However, psychological alterations are not yet known as risk factor for TCR; here is further research needed.

## Conclusion

Heart rate and blood pressure alterations during extraction can be attributed to the occurrence of trigeminocardiac reflex and should be monitored even during simple extraction procedures as the possibility of developing bradycardia and its subsequent syncope exists.

## Competing interest

The authors declare that they have no competing interests.

## Authors’ contributions

AR, RS, MR and BB conceived the study and participated in its design and coordination. AR and RS made substantial contributions to literature review, data acquisition and conception of manuscript. MR conducted statistical analysis. AR and BB drafted and designed the manuscript. RS was involved in revising the manuscript. All authors read and approved the final manuscript.
